# Extracorporeal membrane oxygenation mitigates myocardial injury and improves survival in porcine model of ventricular fibrillation cardiac arrest

**DOI:** 10.1186/s13049-019-0653-z

**Published:** 2019-08-28

**Authors:** Bo Liu, Qiang Zhang, Yong Liang, Yun Zhang, Xiaoli Yuan, Jiyang Ling, Chunsheng Li

**Affiliations:** Department of Emergency Medicine, Beijing Chao-Yang Hospital, Capital Medical University, 8# Worker’s Stadium South Road, Chao-Yang District, Beijing, 100020 China

**Keywords:** Cardiac arrest, Extracorporeal cardiopulmonary resuscitation, Ischemia reperfusion injury, Apoptosis, Cardiac function, Survival

## Abstract

**Introduction:**

Despite decades of improved strategy in conventional cardiopulmonary resuscitation (CCPR), survival rates of favorable neurological outcome after cardiac arrest (CA) remains poor. It is indicated that the survival rate of successful resuscitation of extracorporeal membrane oxygenation (ECMO) is superior to that of CCPR. But the effect of ECMO in heart is unclear. We aimed to investigate whether ECMO produces cardiac protection by ameliorating post-ischemia reperfusion myocardial injury and myocardial apoptosis.

**Methods:**

After undergoing 8-min untreated ventricle fibrillation (VF) and 6-min basic life support, 20 male pigs were ultimately used in this study and randomly divided into two groups: CCPR group (*n* = 10) and extracorporeal CPR (ECPR) group (n = 10). Hemodynamics and blood samples were obtained at baseline and 1, 2, 4, and 6 h during resuscitation. The successfully resuscitated pigs were sacrificed at 6 h after return of spontaneous circulation (ROSC), and the hearts were removed and analyzed under electron microscopy, and immunohistochemistry, quantitative real-time polymerase chain reaction, and immunofluorescence staining assay were performed to evaluate myocardial injury and myocardial apoptosis.

**Results:**

There were no significant differences at basic hemodynamic status between the two groups. The survival rate of ECPR was significantly higher than CCPR group (10/10 [100%] vs. 4/10 [40%], *P* = 0.04). Compared to CCPR group, ECPR group exhibited a better outcome in hemodynamic function. Cardiac function was significantly impaired after ROSC in both groups, but left ventricular ejection fraction (LVEF) was significantly elevated in ECPR group than CCPR group. The expression of myocardial injury biomarkers (CK-MB, cTNI, H-FABP), endothelial injury biomarker (sP-selectin), and cardiac function biomarker (BNP) were remarkably increased after ROSC in both groups, but low levels in ECPR group than in CCPR group. Cardiomyocytes injury was attenuated in ECPR group under transmission electron microscopy (TEM). Typical apoptotic nuclei of cardiomyocytes were significantly reduced and oxidative damage were attenuated in ECPR group.

**Conclusions:**

During prolonged VF-induced CA, ECPR contributes to improving hemodynamics, attenuating myocardial ischemia-reperfusion injury, ameliorating myocardial ultra structure, improving cardiac function, and elevating survival rate by preventing oxidative damage, regulating energy metabolism, inhibiting cardiomyocyte apoptosis.

**Electronic supplementary material:**

The online version of this article (10.1186/s13049-019-0653-z) contains supplementary material, which is available to authorized users.

## Introduction

Despite decades of improved strategy in conventional cardiopulmonary resuscitation (CCPR), survival rates of favorable neurological outcome after cardiac arrest (CA) remains poor [1–6]. The estimated survival rates to hospital discharge with good neurological recovery range from 7.4 to 13.5% for adults with in-hospital CA (IHCA) [2, 6] and 3.2 to 7.3% for those with out-of-hospital CA (OHCA) [1, 3], respectively. With the development of medical technology and advanced devices, extracorporeal CPR (ECPR) by veno-arterial extracorporeal membrane oxygenation (ECMO) is increasingly applied as a rescue therapy for patients resuscitated from CA, and exhibits a higher return of spontaneous circulation (ROSC), improves cardiac function and leads to a favorable neurologically intact survival to hospital discharge compared to CCPR [7–11]. As the target organ of CPR, the recovery of spontaneous beat and function of the heart are very crucial for successful resuscitation after CA. ECPR offers the blood reperfusion and oxygen supply of vital organs during CA and provides a key bridge and time span for therapy decision. However, ECMO in cardioprotection after CA is still lack of evidence. Therefore, this study was designed to investigate the underlying cardioprotection and its mechanism of ECPR in a porcine model of prolonged ventricular fibrillation (VF) CA. We hypothesized that ECPR could attenuate myocardial injury and endothelial damage of post-resuscitation, improve cardiac function, reduce the apoptosis of cardiomyocytes and increase short-term survival rate.

## Methods

This study was approved by the Institutional Animal Care and Use Committee of the Capital Medical University and performed at the Beijing Chao-Yang Hospital Affiliated to the Capital Medical University. All protocols strictly conformed to the National Research Council’s 1996 Guide for the Care and Use of Laboratory Animals.

### Animal preparation

Twenty male pigs aged 11–13 months, with a mean body weight of 35.13 ± 5.57 kg were applied in this study and randomly divided into two groups: CCPR group (treated with CCPR after CA, *n* = 10) and ECPR group (treated with ECMO after CA, n = 10). Animal preparation was discribed in Additional file 1.

### Establishment of ECMO

A 14Fr Biomedicus venous drainage cannula (Medtronic Perfusion Systems, Minneapolis, MN, USA) was inserted into the left femoral artery, and another 12 Fr Biomedicus arterial cannula was advanced into the left internal jugular vein for ECMO. The VA-ECMO circuit was primed by heparinized normal saline (5.0 U/mL) to avoid clotting of the cannulas. The VA-ECMO was driven by a centrifugal pump (ROTAFLOW Console, Maquet) and the water-circulating heat exchanger (Heater-Cooler Unit HCU 30, Maquet) was placed at a temperature of 34 °C. Pump speeds were place at a rate of 50 mL/kg/min at the beginning, and were adjusted to optimize flow and mean arterial blood pressure during ECPR. An initial 500 mL bolus of normal saline and colloidal fluid were infused intravenously, followed by continuous infusion of 200–500 mL/h to compensate for fluid loss.

### Experimental protocol

**Fig. 1:**
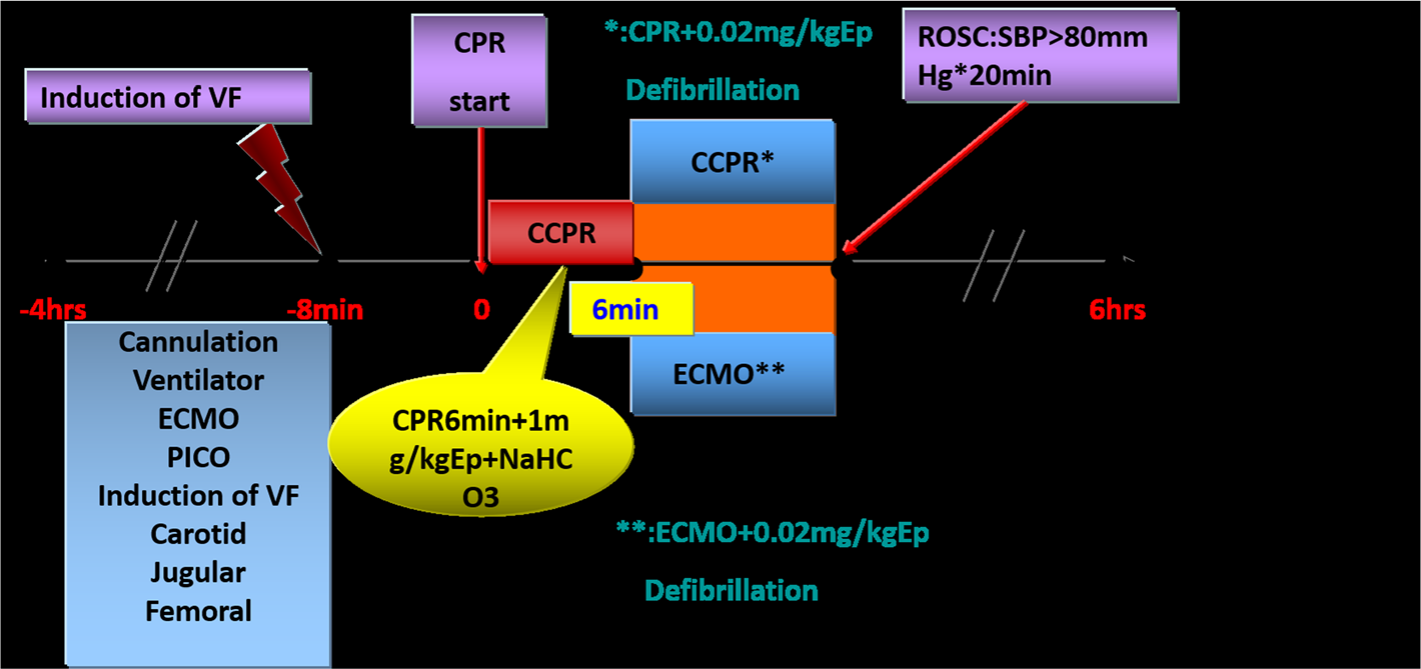
Outline of the experimental protocol. VF, ventricular fibrillation; CCPR, conventional cardiopulmonary resuscitation; ECMO, extracorporeal membrane oxygenation; EP, epinephrine

Experimental protocol was discribed in Additional file 1. The detailed experimental protocol is showed in Fig. 1.

### Outcomes

The primary outcome of successful resuscitation was ROSC, and the secondary endpoints were the successful weaning of ECMO and 6-h short-term survival. Successful weaning was defined as a stable mean aortic pressure (MAP) > 65 mmHg that could be maintained with no ECMO flow.

### Hemodynamic measurements

Hemodynamic variables, including heart rate (HR), MAP, central venous pressure (CVP), CO, CPP using the Swan-Ganz catheter were continuously measured. CPP was defined as the difference between the diastolic aortic pressure and the diastolic right atrial pressure. CO was determined using thermodilution method by injections of 10 mL ice-cold saline, and the CO was calculated as the average value of three consecutive measurements. These parameters were measured at baseline before induction of the VF, and 1 h after ROSC (ROSC 1 h), ROSC 2 h, ROSC 4 h, and ROSC 6 h after ECPR and CCPR.

### Measurements of cardiac function by echocardiography

Echocardiography was performed by an ultrasonic doctor who didn’t participate in the experiment, and measurements were taken at baseline and at ROSC 1, 2, 4, 6 h, respectively. A 2- or 4-chamber long-axis view was obtained using a Hewlett-Packard Sonos 2500 echocardiographic system (Hewlett-Packard, Andover, Mass) with a 5.5/7.5-Hz biplane Doppler transesophageal echocardiographic transducer and a 4-way flexure. Left ventricular end-systolic and end-diastolic volumes were calculated by the disk method (Acoustic Quantification Technology; Hewlett- Packard). These parameters were used to determine left ventricular ejection fraction (LVEF).

### Sample collection

Blood samples were collected at regular time intervals (at baseline, 1, 2, 4, 6 h during resuscitation) from the right femoral artery for measurement of cardiac biomarkers, and were placed in heparinized sterile tubes and centrifuged at 3000 r/min for 10 min, and samples with visible hemolysis were discarded. Plasma was immediately separated and stored at − 80 °C until analysis. Lactate levels were measured by arterial blood gases examined (GEM Premier 3000 blood gas analyzer, Instrumentation Laboratory, Lexington, MA). Myocardial cellular injury was determined with MB isoenzyme of creatine kinase (CK-MB, H197, Nanjing, Jiancheng Bioengineering Institute), cardiac troponin I (cTNI, E019, Nanjing Jiancheng Bioengineering Institute), and heart-type fatty acid-binding protein (H-FABP, Shanghai Renjie Biotechnology Company, Ltd). Cardiac function and myocardial endothelial dysfunction were determined with B-type natriuretic peptide (BNP, H166, Nanjing Jiancheng Bioengineering Institute**)** and sP-selectin (Shanghai Renjie Biotechnology Company, Ltd)**,** respectively. Plasma concentrations of these biomarkers were determined with quantitative sandwich enzyme-linked immunosorbent assay (ELISA) using commercially available kits (R&D).

### Harvest of heart tissue

After the animals were sacrificed at 6 h after ROSC, the hearts were excised, the right ventricle and both atria were removed, and the left ventricle were frozen rapidly by immersion in liquid N_2_ and were stored − 80 °C until requirement of measurements for Na^+^-K^+^-ATPase enzyme activity, Ca^2+^-ATPase enzyme activity, superoxide dismutase (SOD), and malondialdehyde (MDA). Measurements of Na^+^-K^+^-ATPase, Ca^2+^-ATPase, SOD enzyme activity and MDA content were just previously described [12].

### Immunofluorescence staining

Immunofluorescence staining was performed on the fixed cardiac tissue slides using a standard protocol with primary antibodies, including monoclonal anti-Bax, anti-Bcl-2 antibodies and anti-caspase-3 (Beijing Boao Biotechnology Company) at 1:10,000 dilution (Cell Signaling Technology; Danver, USA) and secondary horseradish peroxidase (HRP)-conjugated goat anti mouse antibody at 1:1000 dilution. The staining results were observed under an optical microscopy (CX41; Olympus, Tokyo, Japan).

### Ultrastructural analysis

Some myocardial specimen preserved in 10% formaldehyde and 4% paraformaldehyde were dissected from the left ventricular free walls of porcine hearts and 8 um-thick slices were cut from each tissue block using a cryostat microtome. The ultra structural pathologic changes of the myocardium were observed using light microscopy and TEM (JEM-1010; JEOL, Tokyo, Japan). The pathologic data were assessed by pathologists blinded to the experimental groups.

### Statistical analysis

All data were analyzed using SPSS 19.0 software (SPSS, Chicago, IL, USA). Continuous variables are expressed as mean ± SD. Student’s t-test was used for comparisons between ECPR and CCPR groups. Differences at different time points were assessed by repeated-measures analysis of variance (ANOVA), and *P*-values from post-hoc testing were corrected for multiple comparisons using the Bonferroni correction. Survival analysis was performed using the method of Kaplan and Meier, and comparisons between groups were made using the log-rank test. A two-tailed *P*-value < 0.05 was considered statistically significant.

## Results

### Survival rates

**Fig. 2:**
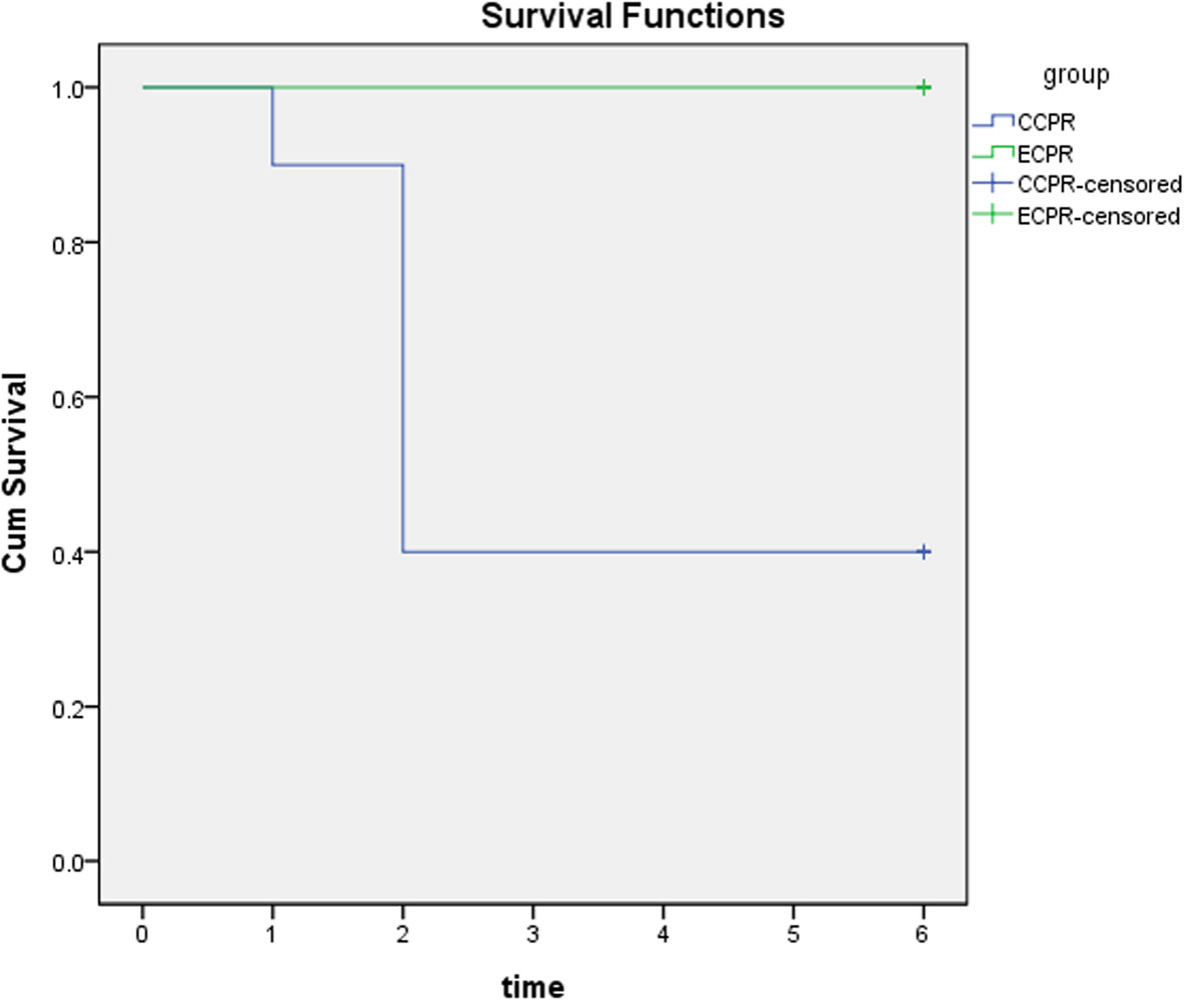
Kaplan-Meier survival curve. There was a significantly higher survival rate in ECPR group compared to CCPR group (*P* < 0.05 by log-rank test)

Twenty animals were successfully resuscitated. In CCPR group, during the following 6-h observation after ROSC, two of ten piglets died at 1 h, another four animals died at 2 h after ROSC, respectively. However, all ten piglets survived in ECPR group after ROSC. There was a significantly higher survival rate in ECPR group by the end of the 6-h experiment period using the Kaplan-Meier survival curve (*P* < 0.05) compared to CCPR group (10/10 [100%] vs. 4/10 [40%]) (Fig. 2).

### Baseline status

Baseline characteristics of the ECPR and CCPR group animals are shown in Table 1. No significant differences were found in baseline weight, HR, CO, MAP, CVP, CPP, hemoglobin level, and lactate level between the two groups (*P* > 0.05).

**Table 1:** Baseline characteristics between ECPR and CCPR groups

Variable	ECPR (n = 10)	CCPR (n = 10)	*P*-value
Weight (kg)	31.70 ± 2.06	31.90 ± 2.51	0.309
HR (bpm)	122.80 ± 9.52	127.60 ± 11.06	0.312
CO (ml/kg/min)	3.78 ± 0.21	3.76 ± 0.26	0.859
MAP (mmHg)	115.80 ± 6.56	116.80 ± 3.52	0.322
CVP (mmHg)	5.40 ± 0.97	5.50 ± 1.08	0.656
CPP (mmHg)	81.80 ± 2.90	83.00 ± 4.50	0.487
Hemoglobin (g/dL)	10.94 ± 0.33	11.02 ± 0.41	0.345
Lactate (mmol/L)	3.03 ± 0.18	2.99 ± 0.17	0.609

*HR* heart rate, *CO* cardiac output, *MAP* mean aortic pressure, *CVP* central venous pressure, *CPP* coronary perfusion pressure

**Fig. 3:**
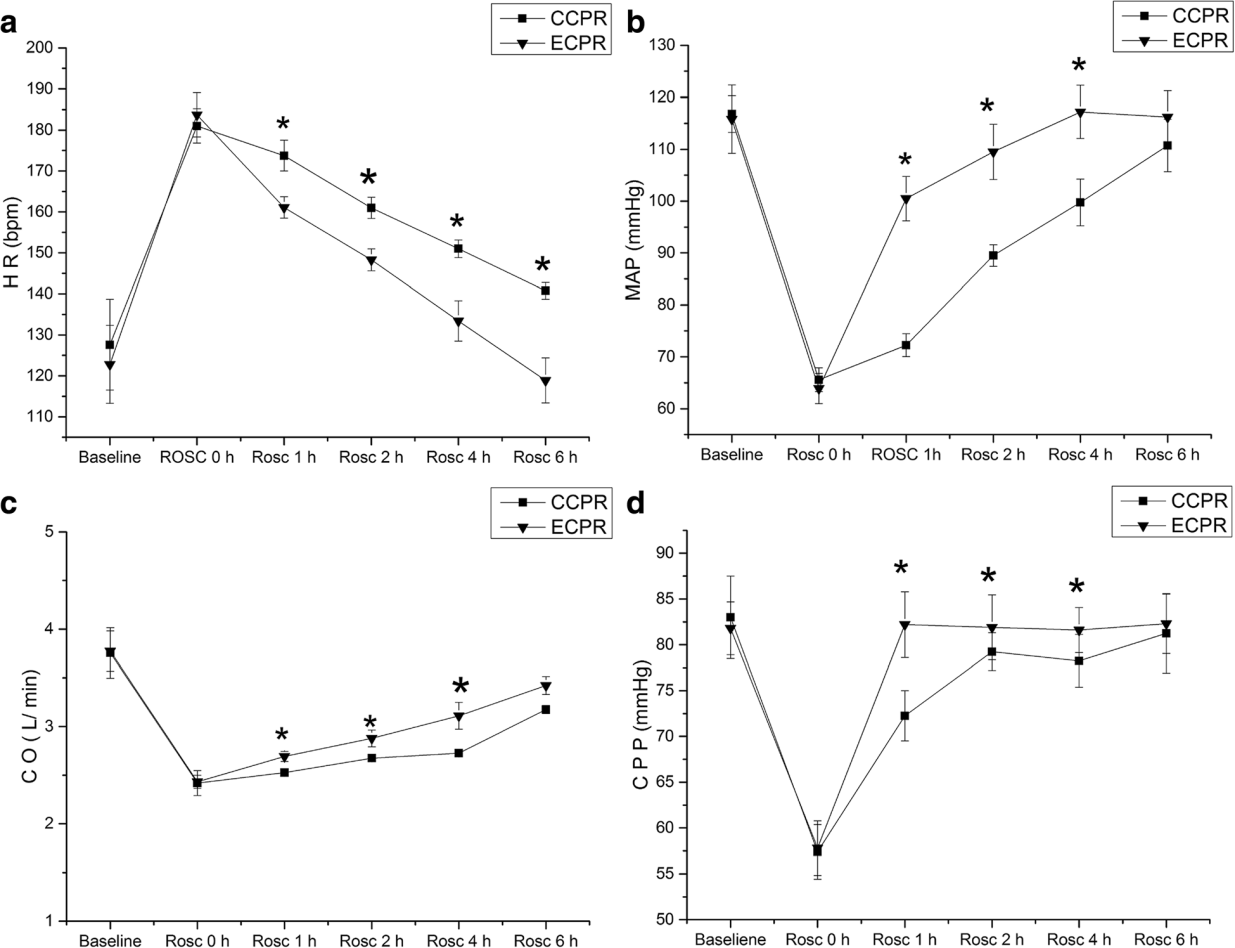
Hemodynamic parameters between ECPR and CCPR groups. HR: heart rate; MAP: mean aortic pressure; CO: cardiac output; CPP: coronary perfusion pressure; CCPR: conventional cardiopulmonary resuscitation; ECPR: extracorporeal cardiopulmonary resuscitation; ROSC: return of spontaneous circulation; The solid triangle indicates ECPR group; The solid square denotes CCPR group; The bar length represents the standard deviation. *P < 0.05 vs. CCPR group

MAP, CO and CPP decreased the minimum, and HR increased the peak at the start-time point of ROSC (ROSC 0) between ECPR group and CCPR group. There were no significant differences in HR [181.0 ± 4.18 vs. 183.7 ± 5.38, *P* = 0.32], MAP [63.9 ± 2.92 vs. 65.6 ± 2.30, *P* = 0.28], CO [2.43 ± 0.07 vs. 2.42 ± 0.13, *P* = 0.79] and CPP [57.8 ± 2.97 vs. 57.4 ± 2.90, *P* = 0.81) between the two groups at ROSC 0. MAP, CO and CPP were significantly higher in ECPR group than in CCPR group at 1 h [MAP, 100.5 ± 4.28 vs. 72.25 ± 2.22; CO, 2.69 ± 0.05 vs. 2.52 ± 0.03; CPP, 82.2 ± 3.58 vs. 72.3 ± 2.75]; 2 h [MAP, 109.5 ± 5.31 vs. 89.5 ± 2.08; CO, 2.87 ± 0.09 vs. 2.68 ± 0.03; and CPP, 81.9 ± 3.54 vs. 79.2 ± 2.60] and 4 h [MAP, 117.2 ± 5.12 vs. 99.75 ± 4.50; CO, 3.11 ± 0.14 vs. 2.73 ± 0.03; CPP, 81.6 ± 2.46 vs. 78.2 ± 2.78] after ROSC, but were not significantly different between the two groups at 6 h after ROSC [MAP, 116.2 ± 5.07 vs. 110.75 ± 5.06, *P* = 0.09; CO, 3.20 ± 0.03 vs. 3.18 ± 0.04, *P* = 0.45; CPP, 82.3 ± 3.23 vs. 81.25 ± 4.35, *P* = 0.62) (Fig. 3b, c, d). However, heart rates were significantly lower at all time points during the 6 h after ROSC in ECPR group compared to CCPR group (Fig. 3a).

### Left ventricle cardiac function evaluation by echocardiography

**Fig. 4:**
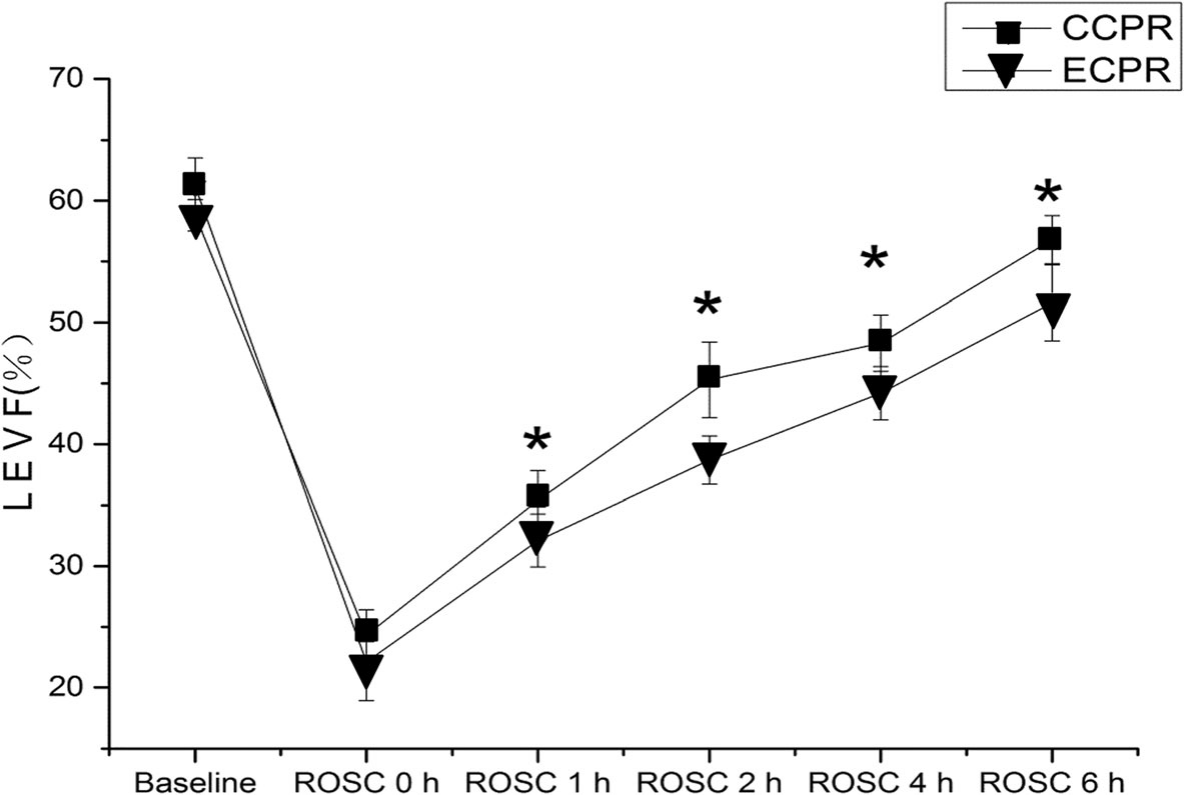
LVEF measured by echocardiography at different measurement times. LVEF: left ventricular ejection fraction, expressed in percentages. *P < 0.05 vs. CCPR group

The dynamic changes of left ventricle ejection fracture (LVEF) over time are showed in Fig. 4. There were no statistical differences for LVEF at baseline between the two groups [61.4 ± 2.22 vs. 60.01 ± 3.40, *P* = 0.29]. LVEF decreased gradually with the incidence of VF, and reached a minimum at the initial time point of ROSC (ROSC 0) in both groups, and then increased significantly with time, verged on the value of baseline at 6 h after ROSC [ROSC 6, 63.5 ± 2.27 vs. 60.25 ± 0.96, *P* = 0.02]. Compared with CCPR group, LVEF were significantly higher in ECMO group at 1 h [43.5 ± 2.25 vs. 32.5 ± 2.08, *P* = 0.001], 2 h [52.3 ± 2.28 vs. 44.25 ± 2.71, *P* = 0.001], and 4 h [56.7 ± 1.64 vs. 52.25 ± 1.26, *P* = 0.001].

### Serum biomarkers levels of myocardial injury and cardiac dysfunction

**Fig. 5:**
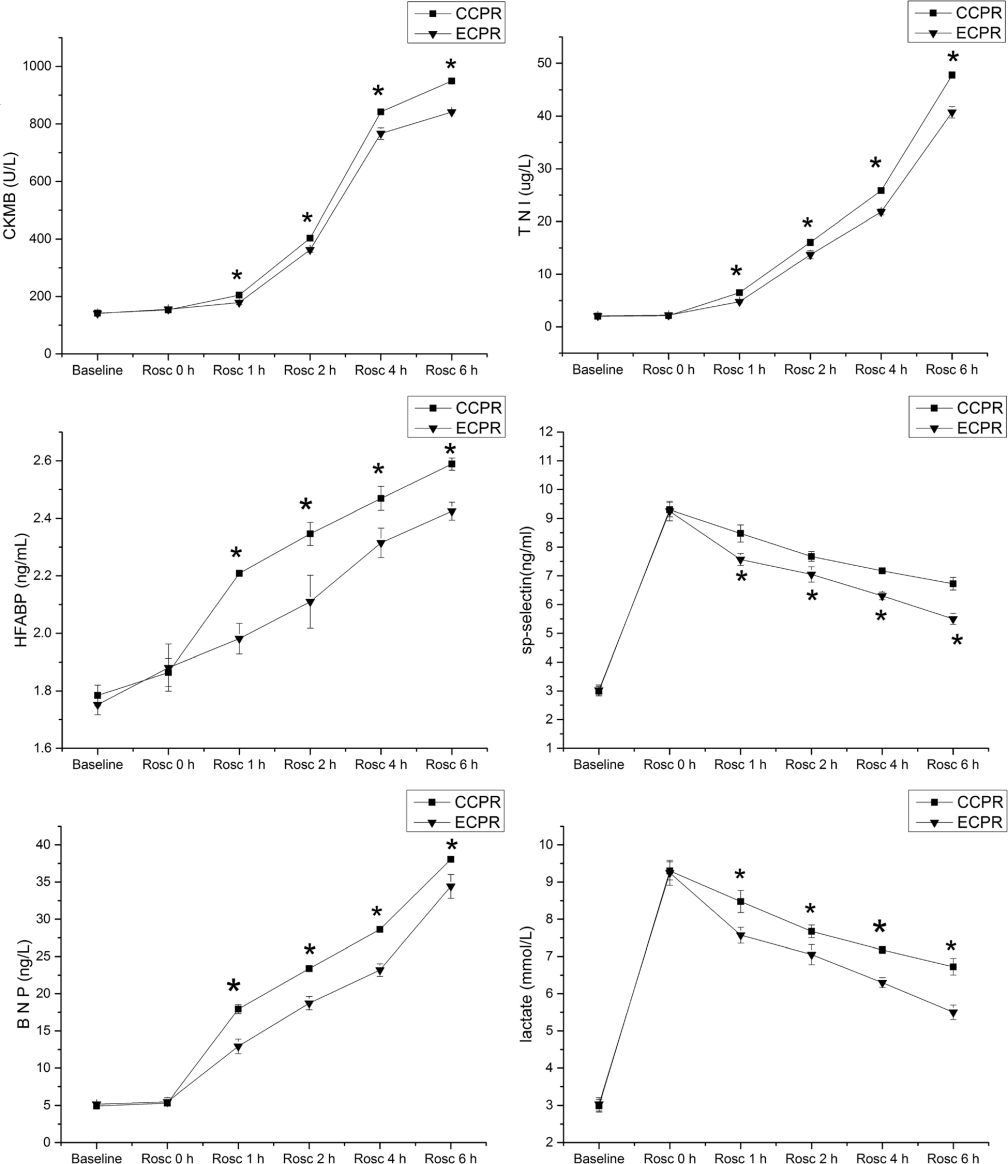
Dynamic changes of serum cardiac biomarkers between ECPR and CCPR groups. cTNI: cardiac troponin I; CK-MB: MB isoenzyme of creatine kinase; H-FABP: heart-type fatty acid-binding protein; sP-selectin: soluble P-selectin; BNP: B-type natriuretic peptide

As showed in Fig. 5, there were no significant differences for the serum levels of cTNI [2.07 ± 0.08 vs. 2.04 ± 0.05, *P* = 0.24]; CK-MB [140.76 ± 1.47 vs. 141.62 ± 1.96, *P* = 0.28]; H-FABP [1.75 ± 0.03 vs. 1.78 ± 0.04, *P* = 0.06] and BNP [5.15 ± 0.3 vs. 4.93 ± 0.24, *P* = 0.09] at baseline and the initial time point of ROSC (ROSC 0) [cTNI, 2.22 ± 0.26 vs. 2.11 ± 0.04; CK-MB, 155.37 ± 4.87 vs. 153.58 ± 2.09; H-FABP, 1.88 ± 0.08 vs. 1.86 ± 0.05; and BNP, 5.48 ± 0.55 vs. 5.30 ± 0.16] between the two groups, and were progressively elevated throughout the study time points after ROSC, reached the peak at 6 h after ROSC, and the levels of cTNI, CK-MB, H-FABP, and BNP were markedly higher in CCPR group compared to ECPR group at 1 h [cTNI, 4.77 ± 0.19 vs. 6.50 ± 0.11; CK-MB, 178.74 ± 7.22 vs. 204.62 ± 0.90; H-FABP, 1.98 ± 0.05 vs. 2.20 ± 0.01; BNP, 12.92 ± 0.99 vs. 17.93 ± 0.60]; 2 h [cTNI, 13.70 ± 0.76 vs. 16.06 ± 0.62; CK-MB, 362.82 ± 8.48 vs. 402.95 ± 2.65; H-FABP, 2.11 ± 0.09 vs. 2.35 ± 0.04; BNP, 23.38 ± 0.36 vs. 18.73 ± 0.89], 4 h [cTNI, 21.80 ± 0.56 vs. 25.84 ± 0.47; CK-MB, 766.5 ± 20.10 vs. 842.10 ± 3.48; H-FABP, 2.32 ± 0.05 vs. 2.47 ± 0.04; BNP, 28.63 ± 0.36 vs. 23.18. ± 0.85] and 6 h after ROSC [cTNI, 40.74 ± 1.07 vs. 47.76 ± 0.43; CK-MB, 841.89 ± 3.54 vs. 949.87 ± 1.78; H-FABP, 2.43 ± 0.03 vs. 2.59 ± 0.02; BNP, 38.05 ± 0.15 vs. 34.42 ± 1.60] (ROSC 1, 2, 4, 6 h) (*P* < 0.05). The sP-selectin concentrations were increased gradually compared to the baseline values before ROSC in the two groups, reached the maximum at ROSC 0 [9.25 ± 0.34 vs. 9.3 ± 0.24] and then decreased with time extension, and reached the minimum at 6 h after ROSC [6.73 ± 0.22 vs. 5.5 ± 0.19]. However, the sP-selectin and lactate concentrations were significantly lower in ECPR group than in CCPR group at 1 h, 2 h, 4 h, and 6 h after ROSC (*P* < 0.05).

### The content of MDA and activities of Na^+^-K^+^-ATPase, Ca^2+^-ATPase and SOD of left ventricle myocardium

The activities of Na^+^-K^+^-ATPase, Ca^2+^-ATPase and SOD of left ventricle myocardium were significantly higher in ECPR group than in CCPR group at 6 h after ROSC. At the same time, the myocardial MDA content was significantly decreased in ECPR group compared to CCPR group (*P* < 0.05; Table 2).

**Table 2:** The content of MDA and the activity of Na^+^-K^+^-ATPase, Ca^2+^-ATPase and SOD in left ventricle tissue at 6 h after ROSC

Parameter	ECPR	CCPR	*P*-value
SOD (NU/mg)	14.00 ± 0.32	11.89 ± 0.55	*P* < 0.05
MDA (umol/g)	15.55 ± 0.53	22.78 ± 0.78	*P* < 0.05
Na^+^-K^+^-ATPase (U)	5.8 ± 0.09	3.19 ± 0.11	*P* < 0.05
Ca^2+^-ATPase (U)	6.33 ± 0.06	4.97 ± 0.15	*P* < 0.05

Values are shown as mean ± SD; *ECPR* Extracorporeal cardiopulmonary resuscitation, *CCPR* Conventional cardiopulmonary resuscitation

### Cardiomyocyte apoptosis

**Fig. 6:**
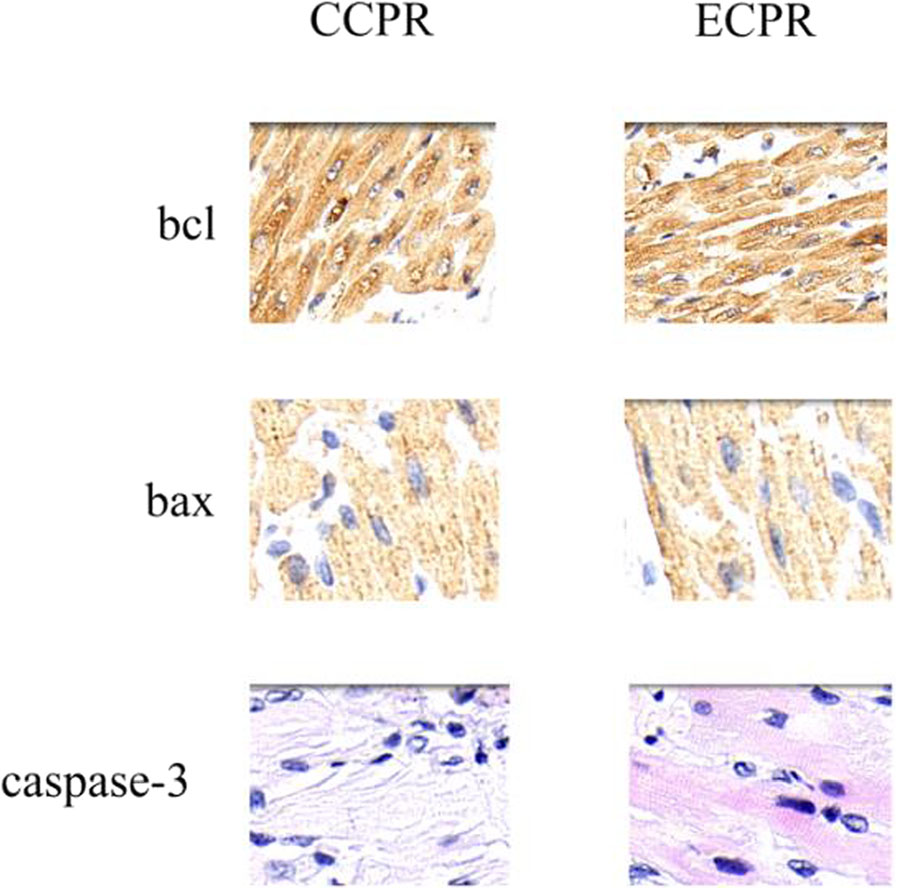
Apoptosis detection in porcine at 6 h after successful resuscitation. Representative images of immunostaining of Bcl-2, bax, and cleaved caspase-3. CCPR: Conventional cardiopulmonary resuscitation; ECPR: Extracorporeal cardiopulmonary resuscitation

Immunofluorescence staining assay was performed to evaluate cardiomyocyte apoptosis and apoptosis was decreased in ECPR groups compared to CCPR group. Furthermore, upregulation of BCL-2 and downregulation of Bax and cleaved caspase-3 expression were detected in ECPR group (Fig. 6).

### Ultrastructural changes in cardiomyocytes

**Fig. 7:**
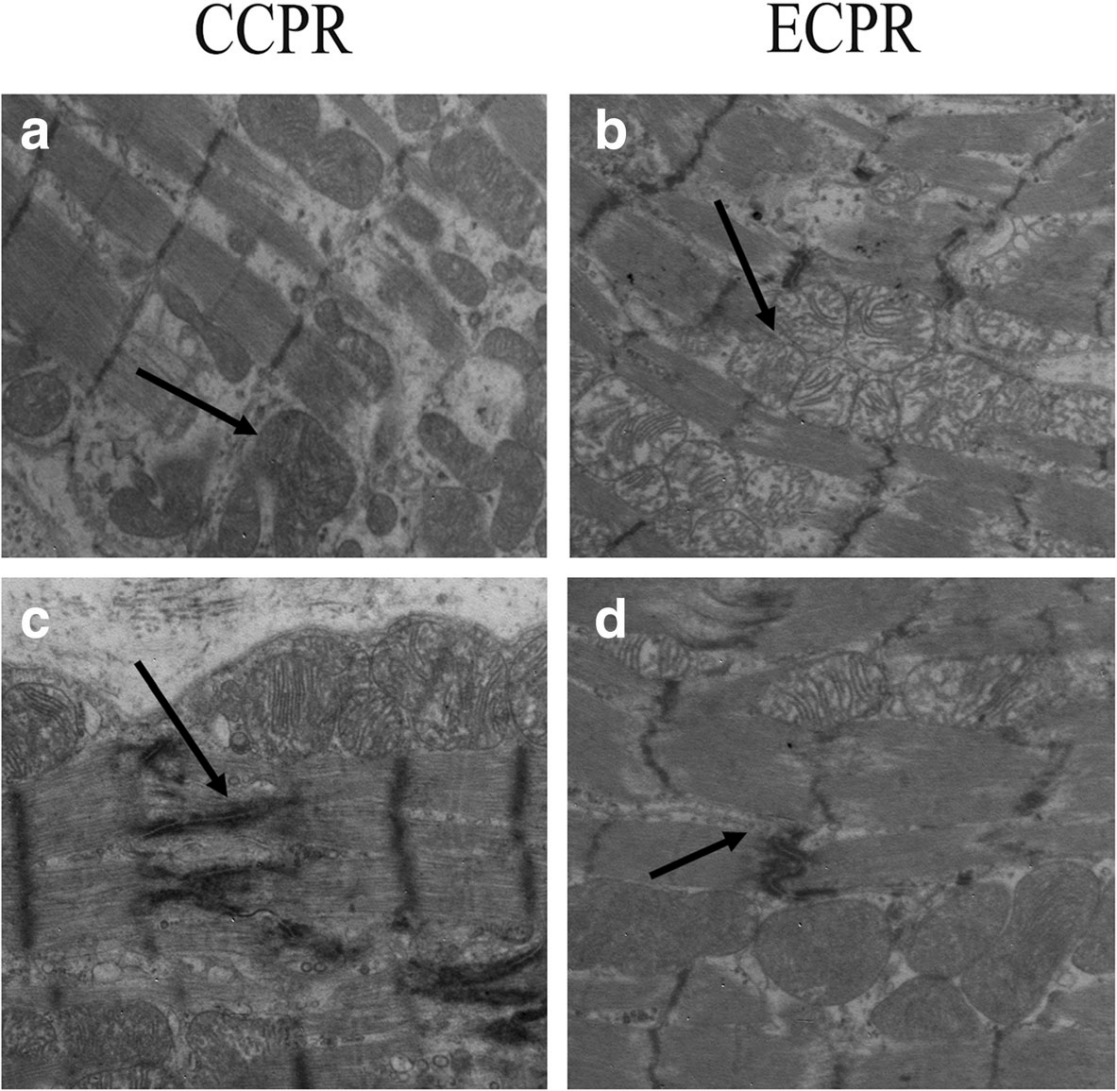
Ultrastructure of the myocardium under TEM at 6 h after successful resuscitation in porcinein CCPR group (**a**, **c**) and in ECPR group (**b**, **d**) (original magnification × 30,000)

Animals were sacrificed after ROSC 6 h, ultramicrostructural changes of cardiomyocytes were observed under TEM. Myocardial fiber and intercalated disk were found to be markedly disordered, broken, or dissolved. Most of the mitochondria were seriously damaged, vacuolar degenerated, mitochondria cristae were vague, arranged irregularly, or disrupted in CCPR group (Fig. 7a, c). However, the morphologic structure of cardiomyocytes included partial nuclear chromatin condensation, crest fracture, and moderate edema in the mitochondria and sarcoplasmic reticula, only exhibiting slight intracellular damage in ECPR group (Fig. 7b, d).

## Discussion

ECPR has been originally used as a therapeutic option during refractory CA since 1976. In the 2010 American Heart Association (AHA) guideline, ECPR has been recommended as an alternative option for patients who have a brief no-flow time and a reversible cause of CA [13]. As the most severe shock state, CA results in the whole-body ischemia-reperfusion, during which the delivery of oxygen and supply of blood are abruptly halted, and a large amount of metabolites are produced and hardly removed [14]. Although conventional CPR may partially reverse this process, CO achieved is still much less than normal [15]. It has been showed that the most important determinant of ROSC during CPR is myocardial blood flow, which is driven by CPP. CPP during CPR has been considered as a leading predictor of successful resuscitation [16, 17]. In our study, pigs that underwent CA between CCPR and ECPR groups presented the significantly decreased MAP, CO, LVEF and CPP, suggesting the severe post-resuscitation myocardial dysfunction (PRMD) and myocardial ischemia. Although the etiology of PRMD is not clear, it is thought to be associated with cardiovascular ischemia/reperfusion injury (IRI), cardiovascular toxicity from excessive levels of inflammatory cytokine activation and catecholamines, and other contributing factors [18]. However, increased microcirculatory blood perfusion, improved cardiac function, reduced oxygen consume of heart, as evidenced by progressively increased MAP, CO, LVEF and lower HR after successful ROSC in ECPR group indicated that ECMO played an important role in the recovery of cardiac function. In addition, a rapid and stable increase for CPP during CPR also ameliorated the myocardial blood perfusion, accelerated the recovery of cardiac function compared to CCPR, which was in line with previous studies [19, 20].

Cardiac biomarkers play a crucial role in the prediction of the severity of myocardial injury, cardiac dysfunction and prognosis in the early stage of post-resuscitation. Currently, serum cTNI, CK-MB and BNP are the most commonly used and sensitive biomarker available for early myocardial injuries and cardiac function [21]. Heart-type *fatty acid-binding protein* (H-FABP) also has been reported to be an early sensitive predictor for myocardial injury and higher plasma H-FABP levels suggest further cardiac events and worse prognosis [22]. Now it is commonly accepted that endothelial dysfunction and inflammation are involved in the physiopathology of CA. It has been showed that P-selectin, as an inflammatory adhesion molecule, its soluble form sP-selectin levels were significantly increased in the early stage of CA [23, 24]. Our findings showed that the levels of cTNI, CK-MB, H-FABP, BNP and sP-selectin were significantly elevated in each group after ROSC, but the concentrations were significantly lower in ECPR group than in CCPR group during resuscitation, suggesting that myocardial injury and endothelial damage were unavoidable after resuscitation, and application of ECMO can attenuate the degree of myocardial injury and endothelial damage, further improve cardiac function. Additionally, further cardioprotection by ECMO was associated with the descendent myocardial loading, decreased myocardial work, and reduced exogenic myocardial impairment by chest compressions.

CA causes inadequate cellular oxygen utilization, and results in an increase of lactate level which reflects abnormal cellular function. Numerous studies have revealed an association between initial serum lactate levels and survival in CA, and reported serum lactate level was an independent prognostic factor of mortality and neurological outcome [5, 25–27]. Hayashida K et al. [28] showed that effective lactate reduction over the first 6 h of post-CA care was implicated in survival and good neurologic outcome independently of the initial lactate level. Oxidative stress plays an important role in the process of IRI. During the CA-associated reperfusion of ischemic myocardium, a large amount of oxygen free radicals (OFRs) overproduced can result in oxidative damage for myocardium. The preserved activity of SOD has capable of clearing OFRs, which displays strong cardioprotective effects [29, 30]. As an end product of lipid peroxidation, MDA gives rise to cellular damage and disruption of cell membranes when tissue antioxidants are exhausted [31]. Na^+^-K^+^-ATPase is an important mediator in the regulation of vasculature tone and contractility, and decreased Na^+^-K^+^-ATPase expression induces cardiomyocytes death, ultimately leading to myocardial dilation, cardiac dysfunction, and even heart failure (HF) [32]. Ca^2+^-ATPase plays the major contribution in cardiomyocyte calcium removal, and is a key regulator of IRI. Similarly, overexpression of Ca^2+^-ATPase preserves cardiac function following IRI, improves cardiac performance and limits cardiac hypertrophy and HF [33]. In our present study, in contrast to CCPR group, ECPR group demonstrated a relatively lower lactate level, MDA content and higher SOD content, Na^+^-K^+^-ATPase and Ca^2+^-ATPase expression during resuscitation, indicating that ECMO may effectively improve tissue perfusion, increase oxygen utility, ameliorate the energy metabolism and reduce oxidative damage of myocardium, and attenuate post-ischemia reperfusion cardiac dysfunction.

Accumulating evidence demonstrate that myocardial ischemia reperfusion after ROSC may induce cardiomyocytes apoptosis [34–36]. Apoptosis results in the reduction of quantities of contractile cardiomyocytes, leading to the decrease of cardiac pump capability, consequently induce hear failure. Bcl-2 (B cell lymphoma gene-2) family plays important roles in the regulation of cardiomyocytes apoptosis. Bcl-2 is the most important antiapoptotic protein, and Bax (Bcl-2-associated protein X) is the most characteristic death-promoting member of the Bcl-2 family. Overexpression of Bcl-2 relative to Bax inhibits apoptosis, and conversely, promotes apoptosis [37]. A family of caspases is another key regulator of apoptotic signaling pathway. Caspase-3 is one of the executioner caspases and is responsible for apoptotic cell death, leading to internucleosomal DNA fragmentation [37]. Aside from contributing to cell death, caspase-3 activation promotes the progressive loss of contractile function in heart by facilitating the degradation of myofibrillar proteins, leading to HF [38]. The present findings demonstrated that ECPR can decrease myocardial apoptosis, as evidenced by upregulation of Bcl-2 in relative to Bax and caspase-3 expression compared to CCPR. In addition, less apoptotic cardiomyocyte were observed under treatment with ECMO. These data indicate that ECMO can reduce IRI-induced cardiomyocye apoptosis, suggesting a key role of ECMO in post-resuscitation myocardial dysfunction. Furthermore, histologic findings further provided the evidence that ECMO can alleviate post-ischemia reperfusion myocardial injury. There a difference between the dosages of epinephrine used between the two groups. The cardioprotectice effect of ECMO in this study could also be driven by saving on cardiotoxic catecholamines.

### Limitations

Our study has some limitations to merit consideration. First, only young healthy pigs without any discernible coexisting disease were used in the experiment, which cannot be compared with humans who suffered from severe disease. The study was performed in animal with healthy hearts. It remains unclear, how ECMO support works in the case of acutely reduced lv dysfunction and a vulnerable heart like ischemic myocardium with baseline elevated filling pressures. Here, ECMO might be even harmful and aggravate myocardial damage due to the burden of the increased afterload. Second, the mechanism how ECMO inhibit myocardial apoptosis is still unknown. Third, the time frame of our study was set at 6 h after ROSC, which may not be sufficient to detect the changes of function and apoptosis of myocardial. Four, in our experiment, ECMO was implanted under controlled conditions before the induction of CA, but in real conditions, we may implant ECMO under ungoing CPR with risk of vascular complications which may lead to delay of ECMO support. Thus, further studies are required to address these limitations.

## Conclusions

Based on these experimental findings, we conclude that ECPR after prolonged VF-induced CA improves hemodynamics, attenuates myocardial injury and endothelial damage after ischemia reperfusion, decreases oxidative damage, reduces cardiomyocyte apoptosis, ameliorates myocardial ultra structure, improves cardiac function, and ultimately elevates the survival rate. This study provides a novel basic animal data for the cardioprotection of ECMO in prolonged VF-induced CA.

## Additional file

Additional file 1: The Arrive Guidelines. (DOC 56 kb)
