# Supplementary material for: Extracorporeal membrane oxygenation mitigates myocardial injury and improves survival in porcine model of ventricular fibrillation cardiac arrest
Source: Scand J Trauma Resusc Emerg Med. 2019 Aug 28;27:82. doi: 10.1186/s13049-019-0653-z (PMC6714103; doi:10.1186/s13049-019-0653-z)
Supplement: Supplementary file 1 — The Arrive Guidelines. (DOC 56 kb) [file 13049_2019_653_MOESM1_ESM.doc]

**Additional file 1: The ARRIVE Guidelines**

**Animal Research: Reporting *In Vivo* Experiments**

|  | **Item** | **Recommendation** |
| --- | --- | --- |
| **TITLE** | **1** | **Extracorporeal membrane oxygenation mitigates myocardial injury and improves survival in porcine model of ventricular fibrillation cardiac arrest** |
| **ABSTRACT** | **2** | **Introduction** Despite decades of improved strategy in conventional cardiopulmonary resuscitation (CCPR), survival rates of favorable neurological outcome after cardiac arrest (CA) remains poor. It is indicated that the survival rate of successful resuscitation of extracorporeal membrane oxygenation (ECMO) is superior to that of CCPR. But the effect of ECMO in heart is unclear. We aimed to investigate whether ECMO produces cardiac protection by ameliorating post-ischemia reperfusion myocardial injury and myocardial apoptosis.  **Methods** After undergoing 8-minute untreated ventricle fibrillation (VF), twenty male pigs were ultimately used in this study and randomly divided into two groups: CCPR group (n = 10) and extracorporeal CPR (ECPR) group (n = 10). Hemodynamics and blood samples were obtained at baseline and 1, 2, 4, and 6 hours during resuscitation. The successfully resuscitated pigs were sacrificed at 6 h after return of spontaneous circulation (ROSC), and the hearts were removed and analyzed under electron microscopy, and immunohistochemistry, quantitative real-time polymerase chain reaction, and immunofluorescence staining assay were performed to evaluate myocardial injury and myocardial apoptosis.  **Results** There were no significant differences at basic hemodynamic status between the two groups. The survival rate of ECPR was significantly higher than CCPR group. Compared to CCPR group, ECPR group exhibited a better outcome in hemodynamic function. Cardiac function was significantly impaired after ROSC in both groups, but left ventricular ejection fraction (LVEF) was significantly elevated in ECPR group than CCPR group. The expression of myocardial injury biomarkers (CK-MB, cTNI, H-FABP), endothelial injury biomarker (sP-selectin), and cardiac function biomarker (BNP) were remarkably increased after ROSC in both groups, but low levels in ECPR group than in CCPR group. Cardiomyocytes injury was attenuated in ECPR group under transmission electron microscopy (TEM). Typical apoptotic nuclei of cardiomyocytes were significantly reduced and oxidative damage were attenuated in ECPR group.  **Conclusions** During prolonged VF-induced CA, ECPR contributes to improving hemodynamics, attenuating myocardial ischemia-reperfusion injury, ameliorating myocardial ultra structure, improving cardiac function, and elevating survival rate by preventing oxidative damage, regulating energy metabolism, inhibiting cardiomyocyte apoptosis. |
| **INTRODUCTION** |  |  |
| - **Background** | **3** | Despite decades of improved strategy in conventional cardiopulmonary resuscitation (CCPR), survival rates of favorable neurological outcome after cardiac arrest (CA) remains poor. The estimated survival rates to hospital discharge with good neurological recovery range from 7.4% to 13.5% for adults with in-hospital CA (IHCA) and 3.2% to 7.3% for those with out-of-hospital CA (OHCA), respectively. With the development of medical technology and advanced devices, extracorporeal CPR (ECPR) by veno-arterial extracorporeal membrane oxygenation (ECMO) is increasingly applied as a rescue therapy for patients resuscitated from CA, and exhibits a higher return of spontaneous circulation (ROSC), improves cardiac function and leads to a favorable neurologically intact survival to hospital discharge compared to CCPR**.** As the target organ of CPR, the recovery of spontaneous beat and function of the heart are very crucial for successful resuscitation after CA. ECPR offers the blood reperfusion and oxygen supply of vital organs during CA and provides a key bridge and time span for therapy decision. However, ECMO in cardioprotection after CA is still lack of evidence. Therefore, this study was designed to investigate the underlying cardioprotection and its mechanism of ECPR in a porcine model of prolonged ventricular fibrillation (VF) CA. We hypothesized that ECPR could attenuate myocardial injury and endothelial damage of post-resuscitation, improve cardiac function, reduce the apoptosis of cardiomyocytes and increase short-term survival rate. |
| - **Objectives** | **4** | This study was designed to investigate the underlying cardioprotection and its mechanism of ECPR in a porcine model of prolonged ventricular fibrillation (VF) CA. |
| **METHODS** |  |  |
| - **Ethical statement** | **5** | This study was approved by the Institutional Animal Care and Use Committee of the Capital Medical University and performed at the Beijing Chao-Yang Hospital Affiliated to the Capital Medical University. |
| - **Study design** | **6** | **RCT** |
| - **Experimental procedures** | **7** | Twenty male pigs aged 11-13 months, with a mean body weight of 35.13 ± 5.57 kg were applied in this study and randomly divided into two groups: CCPR group (treated with CCPR after CA, n = 10) and ECPR group (treated with ECMO after CA, n = 10). |
| - **Experimental animals** | **8** | **Pigs** |
| - **Housing and husbandry** | **9** | All protocols strictly conformed to the National Research Council’s 1996 Guide for the Care and Use of Laboratory Animals. |
| - **Sample size** | **10** | **20** |
| - **Allocating animals to experimental groups** | **11** | **Yes** |
| - **Experimental outcomes** | **12** | Twenty animals were successfully resuscitated. In CCPR group, during the following 6-h observation after ROSC, two of ten piglets died at 1h, 2h after ROSC, respectively. However, all ten piglets survived in ECPR group after ROSC. There was a significantly higher survival rate in ECPR group by the end of the 6-h experiment period using the Kaplan-Meier survival curve (*P* < 0.05) compared to CCPR group |
| - **Statistical methods** | **13** | All data were analyzed using SPSS 19.0 software (SPSS, Chicago, IL, USA). Continuous variables are expressed as mean ± SD. Student’s t-test was used for comparisons between ECPR and CCPR groups. Differences at different time points were assessed by repeated-measures analysis of variance (ANOVA), and *P*-values from post-hoc testing were corrected for multiple comparisons using the Bonferroni correction. Survival analysis was performed using the method of Kaplan and Meier, and comparisons between groups were made using the log-rank test. A two-tailed *P*-value < 0.05 was considered statistically significant. |
| **RESULTS** |  |  |
| - **Baseline data** | **14** | Baseline characteristics of the ECPR and CCPR group animals are shown in Table 1. No significant differences were found in baseline weight, HR, CO, MAP, CVP, CPP, hemoglobin level, and lactate level between the two groups |
| - **Numbers analyzed** | **15** | **20** |
| - **Outcomes and estimation** | **16** | Twenty animals were successfully resuscitated. In CCPR group, during the following 6-h observation after ROSC, two of ten piglets died at 1h, 2h after ROSC, respectively. However, all ten piglets survived in ECPR group after ROSC. There was a significantly higher survival rate in ECPR group by the end of the 6-h experiment period using the Kaplan-Meier survival curve (*P* < 0.05) compared to CCPR group |
| - **Adverse events** | **17** | **Yes** |
| **DISCUSSION** |  |  |
| - **Interpretation/scientific implications** | **18** | **Yes** |
| - **Generalisability/translation** | **19** | **Yes** |
| - **Funding** | **20** | This study was supported by the National Natural Science Foundation of China (No. 81372025) and the 2015 Annual Special Cultivation and Development Project for the Technology Innovation Base of the Beijing Key Laboratory Cardiopulmonary Cerebral Resuscitation (No.Z151100001615056). |
